# Angiopoietin-Like Protein 4 and Insulin-Like Growth Factor-1 Expression in Invasive Breast Carcinoma in Young Women

**DOI:** 10.3390/pathophysiology29010002

**Published:** 2022-01-13

**Authors:** Zaleha Kamaludin, Alaa Siddig, Najib Majdi Yaacob, Alfred K. Lam, Wan Faiziah Wan Abdul Rahman

**Affiliations:** 1 Department of Pathology, School of Medical Sciences, Health Campus, Universiti Sains Malaysia, Kelantan 16150, Malaysia; zalehak@usm.my (Z.K.); alaaabdelaziz@student.usm.my (A.S.); 2 Unit of Biostatistics and Research Methodology, Health Campus, Universiti Sains Malaysia, Kelantan 16150, Malaysia; najibmy@usm.my; 3 School of Medicine, Griffith University, Gold Coast, QLD 4222, Australia; a.lam@griffith.edu.au; 4 Breast Cancer Awareness and Research Unit, Hospital Universiti Sains Malaysia, Kelantan 16150, Malaysia

**Keywords:** angiopoietin-like protein, insulin-like growth factor-1, breast cancer molecular subtypes, breast carcinoma in young women

## Abstract

**Simple Summary:**

Breast cancers arising before the age of 45 years, also known as early-onset breast cancers, have a more aggressive behavior and worse prognosis than late-onset breast cancers. Therefore, there is an urgent need to identify potential therapeutic targets for this group of tumors. In the last decade, angiopoietin-like protein 4 and insulin-like growth factor-1 have attracted attention in clinical research as independent markers of the progression and prognosis of malignancies. In this study, we investigated the expression of both proteins in breast carcinoma tissue from young patients and examined whether their expression can be predicted by clinicopathological parameters.

**Abstract:**

Biomarker identification is imperative for invasive breast carcinoma, which is more aggressive and associated with higher mortality and worse prognosis in younger patients (<45 years) than in older patients (>50 years). The current study aimed to investigate angiopoietin-like protein 4 (ANGPTL4) and insulin-like growth factor-1 (IGF-1) protein expression in breast tissue from young patients with breast carcinoma. Immunohistochemical staining was applied in formalin-fixed, paraffin-embedded samples of breast carcinoma tissue from young patients aged <45 years at the time of diagnosis. Both proteins were expressed in the majority of cases. The highest frequency of positive ANGPTL4 and IGF-1 expression was observed in the luminal A subtype, whereas the HER2-overexpression subtype exhibited the lowest expression frequency for both proteins. There was no significant association between ANGPTL4 (*p* = 0.897) and IGF-1 (*p* = 0.091) expression and molecular subtypes of breast carcinoma. The histological grade was a significant predictor of ANGPTL4 expression (grade 1 vs. grade 3, adjusted odds ratio = 12.39, *p* = 0.040). Therefore, ANGPTL-4 and IGF-1 expressions are common in young breast carcinoma tissue. There is a potential use of them as biomarkers in breast carcinoma.

## 1. Introduction

Breast carcinoma is the most common malignancy affecting women worldwide and the second leading cause of cancer-related death in women following lung cancer [1]. After a diagnosis of breast carcinoma, patient management starts with determining the prognostic and predictive parameters that provide an overview of how the tumor will behave and respond to therapy [2]. Several prognostic and predictive parameters have been described for breast carcinoma, starting with conventional parameters such as the hormone receptors status, histological tumor type, histological grade, tumor size, lymph node involvement, lymphovascular invasion, proliferative markers, ending with molecular markers that aid in predicting clinical outcome [3]. However, no parameters are specific for the managing young age patients (≤45 years), even though early-onset breast cancer is more aggressive and associated with worse outcomes than older-onset breast cancer.

**Figure 1:**
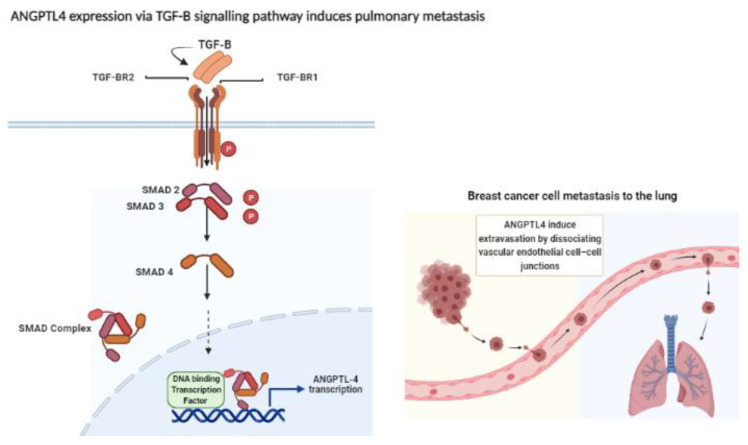
Expression of ANGPTL4 through TGF-B–SMAD signaling enhances the extravasation of the breast cancer cell to the lungs by disrupting of the vascular endothelial cell-cell -junction. Transforming growth factor-B (TGF-B) ligand binds to transforming growth Factor-B receptor I (TGFBR1) and transforming growth factor-B II (TGFBR2). TGFBR2 phosphorylates (P) TGFBR1, which in turn activates SMAD2 and SMAD3 via phosphorylation. Activated SMAD2 and SMAD3 form complex with SMAD4. The complex translocates into the nucleus where it interacts with other DNA-binding transcription factors, co-activators as well as co-repressors. Following this, the complex binds to the promoter regions of TGF target genes and regulates their transcription. The overexpression of angiopoietin-like 4 (ANGPTL-4) induces the extravasation of breast cancer cell by disrupting the vascular endothelial cell-cell junction, Figure created with BioRender.com [9,11].

Angiopoietin-like protein 4 (ANGPTL4) is a secretory glycoprotein belonging to the angiopoietin family. It is expressed in several tissues including liver, adipose, pancreatic, placenta, kidney, intestinal, and ischemic tissue. ANGPTL4 has important roles in lipid and glucose metabolism as well as angiogenesis [4,5]. In 2011, Nakayama and colleagues suggested that ANGPTL4 contributes to progression, venous invasion, and distant metastasis in human colorectal cancer [6]. In 2013, Adhikary et al. reported that the repression of ANGPTL4 gene transcription resulted in the inhibition of breast cancer cell invasion in vitro, suggesting the potential role of ANGPTL4 as a therapeutic target for breast cancer [7]. Furthermore, a recent study reported that higher tissue expression of ANGPTL4 was correlated with breast tumor progression and distant metastasis, as well as shorter overall survival and disease-free survival [8]. The mechanism through which ANGPTL-4 promotes breast cancer progression was described by Padua et al., where they found that the cytokine transforming growth factor-B (TGF-B) induces breast cancer cell metastasis to the lungs, and the key molecule in this process was the overexpressed ANGPTL-4 through SMAD signaling pathway [9] (Figure 1).

In contrast to these findings, Cai et al. found that overexpression of ANGPTL4 inhibits triple-negative breast cancer (TNBC) cell adhesion, migration, and invasion in vitro, suggesting that the enhancement of ANGPTL4 expression can prevent progression in TNBC cells [10].

**Figure 2:**
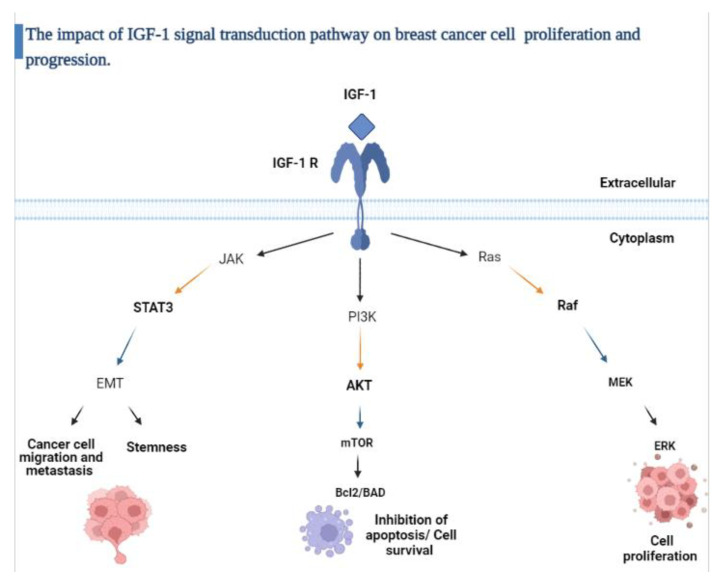
Schematic overview of the IGF-1 signaling pathway.

Another important marker in breast cancer is insulin-like growth factor-1 (IGF-1), which is a polypeptide encoded by the IGF-1 gene located on chromosome 12. IGF-1 is produced mainly in the liver under direct stimulation by growth hormone [12]. Under normal physiological conditions, IGF-1 induces mitosis and inhibits apoptosis. In contrast, under pathological conditions, it is well established that IGF-1 functions as a potent mitogen that contributes to tumor progression, increasing metastatic potential and resistance to apoptosis induced by cytotoxic drugs. In prior studies, IGF-1 overexpression was associated with aggressive tumors and poor prognosis [13,14]. In breast cancer, IGF-1 regulates cell growth through stimulation of the insulin growth factor receptor (IGF-1R), which consequently phosphorylates either insulin receptor substrate-1 (IRS-1) or insulin receptor substrate-2 (IRS-2). IRS-1/2 activates downstream signaling proteins such as PI3K/AKT/mTOR and Ras/Raf/MAPK, and this role has been documented in all molecular subtypes of breast cancer (Figure 2) [15].

The insulin growth factor-1 (IGF-1) signaling axis is activated by the binding of (IGF-1) to the insulin growth factor-1 receptor (IGF-1R); this binding activates several downstream molecular pathways including PI3K/AKT which inhibits apoptosis, increases protein synthesis and glucose metabolism, RAS/MAPK that promotes cell proliferation and JAK/STAT which induces epithelial- mesenchymal transition (EMT) and cancer cell stemness. Figure created with BioRender.com [12,16,17].

In the present study, we investigated the protein expression of ANGPTL4 and IGF-1 in breast carcinoma tissue from young age patients and determined whether there is an association between the expression of both proteins and the breast carcinoma molecular subtype and if the expression of both proteins can be predicted by tumor clinicopathological characteristics.

## 2. Materials and Methods

### 2.1. Study Design, Participants, and Specimen

This cross-sectional study was carried in the Department of Pathology—Hospital Universiti Sains Malaysia, Kelantan, Malaysia. All patients who were histopathologically diagnosed with breast carcinoma between 1 January 2007 and 31 December 2017 and were ≤45 years at the time of diagnosis were included. Patients with unavailable tissue blocks or who had a tissue block but with inadequate material for immunohistochemical staining were excluded. Additionally, patients who received neoadjuvant therapy before surgery were also excluded. The study included 75 patients who fulfilled the study inclusion criteria. Demographic and clinicopathological characteristics of patients including age, specimen type, tumor size, histopathological type, tumor grade, lymph node status, expression of estrogen receptor (ER), progesterone receptor (PR), HER2 by immunohistochemistry and HER2 status by the dual-color dual-hapten brightfield in situ hybridization method (DDISH) were retrieved from the electronic medical records of the department of pathology and patient case records.

### 2.2. Immunohistochemical Staining and Scoring System

Briefly, tissue sections of 3–4 µm thickness were cut from formalin-fixed paraffin-embedded tissue blocks. Following this, tissue sections were deparaffinized by xylene and rehydrated through a series of graded alcohol. The slides were then placed in an ethylenediaminetetraacetic acid- containing buffer in a pressure cooker for 3 min; the purpose of this step is to retrieve the antigen. Following this, slides were immersed in 3% hydrogen peroxide for 5 min to block endogenous peroxidase activity. Subsequently, for each case one slide was incubated with a rabbit polyclonal ANGPTL4 antibody (1:500; ab196746, Abcam, Cambridge, UK). The second slide was incubated with rabbit polyclonal IGF-1 antibody (1:500; ab9572, Abcam UK) for one hour at room temperature. 3, 3′-diaminobenzidine tetrahydrochloride (DAB) from (DAKO, Glostrup, Hovedstaden, Denmark) was used to visualize the antigen–antibody complex. Esophageal squamous cell carcinoma tissue was used as positive controls for the ANGPTL4 antibody, and placenta tissue was used as positive controls for the IGF-1 antibody, as recommended by the manufacturer. The same tissues were also used as negative controls by omitting the primary antibody incubation step from the protocol.

We used a semi-quantitative scoring system for both antibodies. The scoring system determined the staining intensity and percentage of positive malignant cells. The staining intensity of malignant cells was measured on a scale from 0 to 3 as shown in Table 1. To obtain the immunostaining score, the scale of staining intensity and the percentage of positive malignant cells were multiplied, a total score of ≥4 was defined as a positive expression, whereas a total score of <4 indicated negative expression [18].

Nuclear immunoreactivity of control tissue was necessary for the ANGPTL4 antibody, whereas cytoplasmic immunoreactivity of control tissue was required for the IGF-1 antibody. All slides were scored independently by two well-trained pathologists who were blinded to the clinicopathological data.

### 2.3. Statistical Analysis

Double data entry was performed independently using Microsoft Excel 2010 by two researchers. Data analysis was conducted using R software version 4.0.3 in the R studio environment. Descriptive statistics were performed to describe the sociodemographic as well as the clinicopathological characteristics of the study participants. For age, mean and standard deviation (SD) was presented, for all other variables, frequency (*n*) and percentage (%) were presented.

Associations of ANGPTL4 and IGF-1 expression with molecular subtypes of breast carcinoma were assessed using Pearson’s chi-square test and Fisher’s exact test. Multiple logistic regression analysis was conducted to determine the clinicopathological predictors of ANGPTL4 and IGF-1 expression. Data were presented as adjusted ORs, 95% CIs, and *p*-values.

## 3. Results

**Figure 3:**
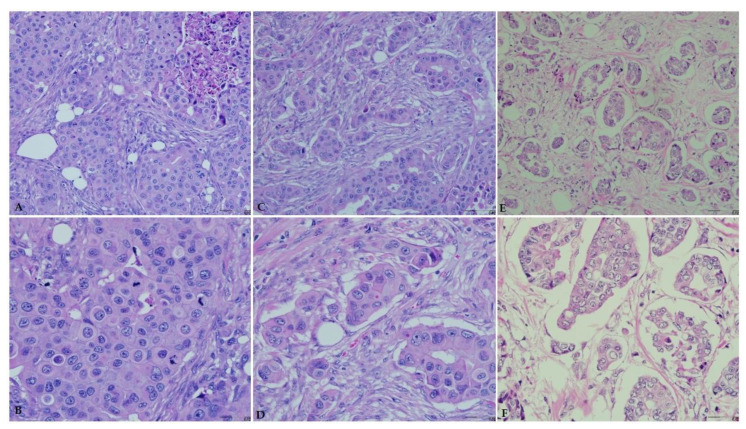
Breast carcinoma histological grade according to the Bloom–Richardson grading system. Hematoxylin and eosin staining (×20 and ×40). (**A**) High grade (grade III, ×20). (**B**) High grade (grade III, ×40). (**C**) High grade (grade II, ×20). (**D**) High grade (grade II, ×40). (**E**) Low grade (grade I, ×20). (**F**) Low grade (grade I, ×40).

The ages of the 75 patients included in this study range between 23 and 44 years with a mean age of 37 years (SD, 5.37 years). Based on the modified Bloom–Richardson grading system, this study included 10 (13.3%) and 65 (86.7%) patients with low-grade (grade I) and high-grade (grades II or III) breast carcinoma, respectively (Figure 3). In addition, 42 (56.0%) and 36 (48.0%) of patients were positive for ER and PR, respectively. Majority of the patients *n* = 40 (53.3%) were HER2-negative (score 0 or 1), whereas 13 (17.3%) patients were equivocal (score, 2) and 22 (29.3%) patients were HER2-positive (score, 3). The most common molecular subtype was Luminal A accounting for 29 (38.7%) cases, followed by triple-negative, luminal B, and HER2-overexpression 20 (26.7%), 15 (20%), and 11 (14.7%), respectively. Further details of the cases clinicopathological characteristics are presented in (Table 2).

### 3.1. Immunohistochemical Expression of ANGPTL-4 and IGF-1

**Figure 4:**
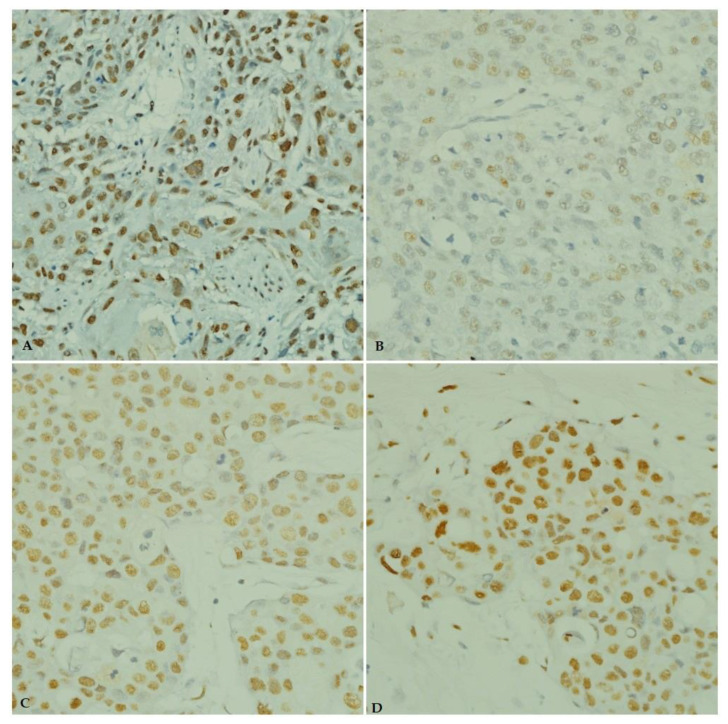
Immunohistochemical staining of ANGPTL4 in breast carcinoma tissue from young patients. (**A**) ANGPTL4 positive control (esophageal squamous cell carcinoma, ×40). (**B**) Weak ANGPTL4 immunostaining. (**C**) Moderate ANGPTL4 immunostaining (×40). (**D**) Strong ANGPTL4 immunostaining (×40).

**Figure 5:**
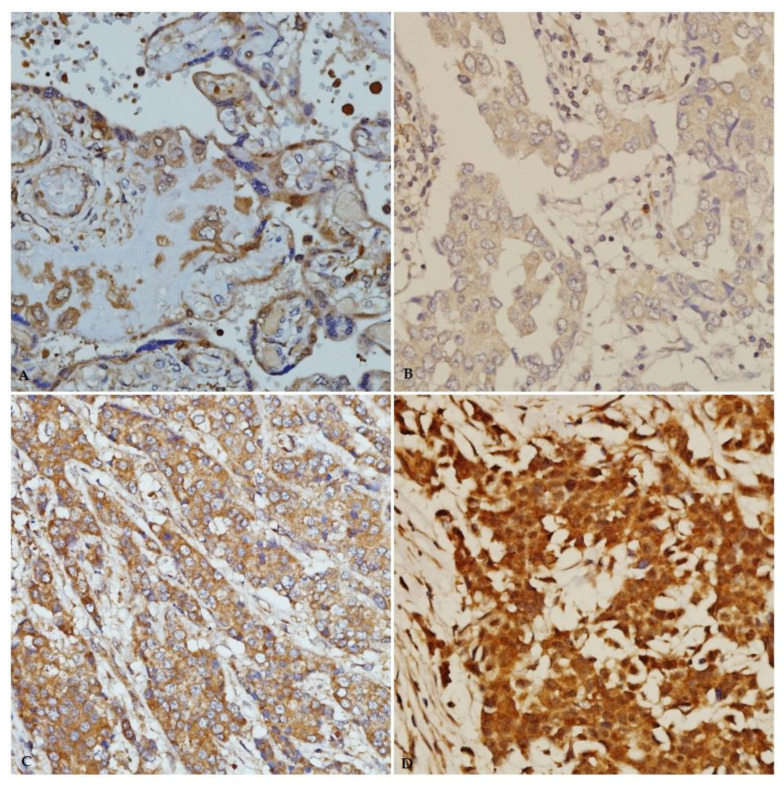
IGF-1 immunohistochemical staining in breast carcinoma tissue from young patients. (**A**) Immunostaining of positive-control placenta tissue (×40). (**B**) Weak IGF-1 immunostaining (×40). (**C**) Moderate IGF-1 immunostaining (×40). (**D**) Strong IGF-1 immunostaining (×40).

As shown in Table 3, the majority of the cases showed immunoreactivity for ANGPTL4 and IGF-1 antibodies in 50 (66.7%) and 67 (89.3%) cases, respectively. Figure 4 and Figure 5 illustrates the immunostaining and scoring of ANGPTL4 and IGF-1 antibodies, respectively.

### 3.2. Associations of ANGPTL-4 and IGF-1 Expression with Breast Carcinoma Molecular Subtypes

The frequency of ANGPTL4 immunoreactivity varied among different molecular subtypes of breast carcinoma, higher frequency was observed in the luminal A subtype (*n* = 19 (38.0%)), followed by triple-negative (*n* = 14 (28.0%)) and luminal B (*n* = 9 (18.0%)), while the lowest rate of ANGPTL4 immunoreactivity was observed in the HER2-overexpression subtype (*n* = 8 (16.0%)). However, the chi-square test did not detect a significant association between ANGPTL4 expression and specific molecular subtypes of invasive breast carcinoma in young women (*p* = 0.897) (Table 4).

Similarly, frequency of IGF-1 immunoreactivity among different molecular subtypes of breast carcinoma was mimicking that observed in ANGPTL4. A high rate of IGF-1 immunoreactivity was observed in the luminal A subtype (*n* = 27 (40.3%)) and the lowest rate of IGF-1 immunoreactivity was found in the HER2-overexpression subtype (*n* = 10 (14.9%)), whereas both the luminal B and triple-negative subtypes showed equal rates of IGF-1 immunoreactivity (*n* = 15 (22.4%)). However, there was no significant association between IGF-1 expression and molecular subtypes (*p* = 0.091) (Table 5).

### 3.3. Clinicopathological Predictors of ANGPTL-4 and IGF-1 Expression

#### 3.3.1. Clinicopathological Predictors of ANGPTL4 Expression

Histological grade, tumor size, lymph node, ER and PR status, HER2 expression, and molecular subtype were included in multiple logistic regression analysis. Table 6 presents the clinicopathological characteristics of patients based on the ANGPTL4 status. Likewise, patients with an unknown tumor size or unknown lymph node status were excluded from the analysis.

In multivariate analysis, patients with grade 1 tumors had 12.39-fold higher odds of positive ANGPTL4 expression than patients with grade 3 tumors following adjustment for all other clinicopathological characteristics (adjusted OR = 12.39, *p* = 0.040; Table 7).

#### 3.3.2. Clinicopathological Predictors of IGF-1

Similar variables as described previously were included in multivariate analysis to identify the clinicopathological predictors of IGF-1 expression. Table 8 presents the patients’ clinicopathological characteristics based on the IGF-1 status.

In multiple logistic regression analyses, the OR could not be computed for histological grade 1 compared to grade 3 because none of the patients with grade 1 tumors was negative for IGF-1 expression. None of the clinicopathological characteristics were predictive of IGF-1 expression (Table 9).

## 4. Discussion

The purpose of the current study was to investigate the protein expression of ANGPTL4 and IGF-1 in primary breast carcinoma tissue and its association with breast cancer molecular subtypes in patients younger than 45 years. Furthermore, we assessed whether the expression of both proteins can be predicted by various clinicopathological parameters.

Shafik et al. and Cai et al. reported expression of ANGPTL4 in the cytoplasm of breast carcinoma cells [10,19]. However, in this study we observed ANGPTL4 expression in the nucleus; we attributed this variation to the dual location of ANGPTL4 in the cell, based on information available in The Human Protein Atlas and GeneCards, ANGPTL4 localized in nucleoplasm as well as vesicles [20]. More than half of patients in the present study displayed immunoreactivity for ANGPTL4 (67%), this in concordance with previous studies investigated ANGPTL4 expression in breast and prostate cancer tissues [19,21]. This finding was expected because ANGPTL4 expression is upregulated in the hypoxic microenvironment, which is a hallmark of most solid tumors including breast cancer [22,23]. Using multiple logistic regression analysis, all clinicopathological parameters, including tumor size, lymph node status, ER and PR status, HER2 expression, and molecular subtype, failed to predict the expression of ANGPTL4. This is in line with a previous study that found no relationships between ANGPTL4 expression and clinicopathological parameters in patients with TNBC [10]. However, when we carried multivariate analysis, patients with grade 1 tumor had 12.39-fold higher odds of positive ANGPTL4 expression than those with grade 3 tumors after adjustment for all other clinicopathological characteristics (adjusted OR = 12.39, *p* = 0.040), this is in contrast to Shafiq et al.’s findings where ANGPTL4 mRNA transcript and serum levels were significantly higher in high grade breast carcinoma samples compared to low grade and control samples. When we compared our data with this study the only difference is the age of breast cancer patients [19]. Thus, further study is needed to elucidate the mechanism behind the variation of ANGPTL-4 expression among different disease grades mainly in young age patients. Findings in this area are controversial. Although several studies concluded that ANGPTL4 expression is associated with tumor progression and metastasis [19,24], many studies implicated ANGPTL4 in the inhibition of tumor angiogenesis and metastasis [25,26]. This conflict of function was explained by Tan et al., who found that ANGPTL4 function was context- and tissue type-dependent [27].

The majority of patients in the current study (89%) exhibited positive staining for IGF-1. This percentage was comparable to that reported by Shiratsuchi et al. in colorectal cancer tissue (80%) [28]. This high frequent expression might be attributed to the role of the growth hormone/IGF-1 axis in oncogenesis and progression of cancer. Specifically, higher plasma levels of IGF-1 were reported previously to be associated with malignancy, whereas deficiencies of growth hormone and IGF-1 were related to the absence of cancers [29]. Most patients with positive staining for IGF-1 had a luminal A subtype, while lower expression was seen in patients with Triple negative subtype. This finding in line with a previous genomic study that found that breast tumors which were known to be well differentiated and have favorable outcomes such as luminal A and normal subtypes exhibited higher expression of the IGF-1 ligand genomic signature, whereas the basal-like subtype which have poor outcomes have lower expression of the IGF-1 ligand genomic signature; furthermore, the study reported results of network analysis, where breast cancer group with high IGF-1 ligand signature shows downregulation in component of proliferation pathways (AKT/MAPK) and upregulation of component of differentiation pathways (adipocyte growth factors, PPAR-gamma), this explains the favorable outcome of tumors with a high IGF-1 ligand signature [30]. Another explanation of the higher expression of IGF-1 in luminal A subtype tumors is the strong crosstalk between ER and IGF-1 signaling pathways [31]. It is well established that components of the IGF-1 signaling pathway are regulated by estrogen [32,33]. Although IGF-1 expression was more frequent in the luminal A subtype and less frequent in the Triple negative subtype, fisher’s exact test failed to find an association between IGF-1 expression and young age breast carcinoma molecular subtypes. Moreover, no clinico–pathological parameter was predictive of IGF-1 expression in breast cancer tissue in young patients.

## 5. Conclusions

In this cross-sectional study we investigated the expression of ANGPTL-4 and IGF-1 in primary breast carcinoma tissue samples from young age patients ≤45 years. Both proteins were expressed by the majority of patients. However, no association was detected between expression of both proteins and breast carcinoma molecular subtype. However, all clinicopathological parameters investigated in the current study failed to predict the tissue expression of both proteins. ANGPTL-4 showed significant higher expression in tumors with lower histopathological grades compared to tumors of higher grades. Further study with a larger sample size may verify our findings.

## Figures and Tables

**Table 1:** Scoring system of ANGPTL4 and IGF1 expression.

% of Positive Cells	Intensity Scoring	Score (0–12)	IRS Classification
0 = No positive cells	0 = No color	0–1 = Negative	0 = Negative
1 ≤0%	1 = Mild reaction	2–3 = Mild	1 = Positive, weak
2 = 10–50%	2 = Moderate reaction	4–8 = Moderate	2 = Positive, intermediate
3 = 51–80%	3 = Intense reaction	9–12 = Strong positive	3 = Positive, strong
4 ≥80%			
Percentage of positive cells X intensity staining = score (0–12)			

IRS, Immunoreactivity scoring system.

**Table 2:** Clinicopathological characteristics of the patients (*n* = 75).

Variables	Mean (SD)	*n* (%)
**Age**	37.24 (5.37)	
Age group		
20–29		6 (8)
30–39		38 (50.7)
40–44		31 (41.3)
**Race**		
Malay		70 (93)
Chinese		2 (3)
Others		3(4)
**Specimen Type**		
Biopsy		29 (39)
Wide local excision		3 (4)
Mastectomy		43 (57)
**Tumor Size**		
Biopsy		29 (39)
<5 cm		18 (24)
≥5 cm		28(37)
**LN Status**		
Positive		55 (73.7)
Negative		17 (22.6)
Unknown		3 (4)
**Histology Subtype**		
Invasive carcinoma, NST		70 (93.3)
Invasive lobular carcinoma		1 (1.3)
Metaplastic carcinoma		3 (4)
Mucinous carcinoma		1 (1.3)
**Histological Grade**		
I		10 (13.3)
II		39 (52)
III		26 (34.7)
**ER Status**		
Positive		42 (56)
Negative		33 (44)
**PR Status**		
Positive		36 (48)
Negative		39 (52)
**HER2 Status**		
0		21 (28)
1		19 (25.3)
2		13 (17.3)
3		22 (29.4)
**Molecular Subtype**		
Luminal A		29 (38.7)
Luminal B		15 (20)
HER overexpression		11 (14.7)
Triple-negative		20 (26.7)

**Table 3:** Expression of ANGPTL-4 and IGF-1 in young patients with breast cancer (*n* = 75).

Expression	N	Proportion (95% CI)
**ANGPTL-4**		
Negative	25	
Positive	50	66.7 (54.8, 77.1)%
**IGF-1**		
Negative	8	
Positive	67	89.3 (80.1, 95.3)%

**Table 4:** Association of ANGPTL-4 expression with breast carcinoma molecular subtypes in young patients (*n* = 75).

Molecular Subtype	ANGPTL-4 Expression		χ2 Statistic (df)	*p*-Value ^1^
	Negative *n* (%)	Positive *n* (%)		
Luminal A	10 (40.0)	19 (38.0)	0.60 (3)	0.897
Luminal B	6 (24.0)	9 (18.0)		
HER-2 overexpression	3 (12.0)	8 (16.0)		
Triple-negative	6 (24.0)	14 (28.0)		

^1^ Chi-square test *p*-value.

**Table 5:** Association of IGF-1 expression with breast carcinoma molecular subtypes in young patients (*n* = 75).

Molecular Subtype	IGF-1 Expression		*p*-Value ^1^
	Negative (*n* = 8)	Positive (*n* = 67)	
Luminal A	2 (25.0)	27 (40.3)	0.091
Luminal B	0 (0.0)	15 (22.4)	
HER-2 overexpression	1 (12.5)	10 (14.9)	
Triple-negative	5 (62.5)	15 (22.4)	

^1^ Fisher’s exact test *p*-value.

**Table 6:** Patients’ clinicopathological characteristics based on the ANGPTL4 status (*n* = 75).

Clinicopathological Characteristics	ANGPTL4 Expression	
	Negative (*n* = 25)	Positive (*n* = 50)
**Histological Grade**		
Grade 1	1 (4.0)	9 (18.0%)
Grade 2	14 (56.0)	25 (50.0%)
Grade 3	10 (40.0)	16 (32.0%)
**Tumor Size**		
<5 cm	11 (44.0)	19 (38.0%)
>5 cm	14 (56.0)	29 (58.0%)
Unknown	0 (0.0)	2 (4.0%)
**Lymph Node**		
Negative	4 (16.0)	13 (26.0)
Positive	21 (84.0)	34 (68.0)
Unknown	0 (0.0)	3 (6.0)
**ER Status**		
Positive	10 (40.0)	23 (46.0)
Negative	15 (60.0)	27 (54.0)
**PR Status**		
Positive	13 (52.0)	26 (52.0)
Negative	12 (48.0)	24 (48.0)
**HER2 Status**		
Negative (0, 1+, 2+)	19 (76.0)	34 (68.0)
Positive (3+)	6 (24.0)	16 (32.0)
**Molecular Subtype**		
Non-triple–negative	19 (76.0)	36 (72.0)
Triple-negative	6 (24.0)	14 (28.0)

**Table 7:** Multiple logistic regression analysis of the clinicopathological predictors of ANGPTL4 expression (*n* = 75).

Clinicopathological Predictors	Adjusted OR (95% CI)	*p*-Value
**Histological Grade**		
Grade 1	12.39 (1.54, 277.51)	0.040
Grade 2	1.79 (0.56, 6.20)	0.334
Grade 3	1	
**Tumor Size**		
<5 cm	1	
>5 cm	2.11 (0.57, 8.34)	0.268
**Lymph Node**		
Negative	1	
Positive	0.35 (0.06, 1.60)	0.198
**ER Status**		
Positive	1	
Negative	0.92 (0.12, 6.98)	0.930
**PR Status**		
Positive	1	
Negative	0.65 (0.12, 3.43)	0.607
**HER2 Status**		
Negative (0, 1+, 2+)	1	
Positive (3+)	3.47 (0.80, 18.00)	0.111
**Molecular Subtype**		
Non-triple–negative	1	
Triple-negative	3.83 (0.49, 32.75)	0.204

Model fitness for multiple logistic regression analysis: Hosmer–Lemeshow chi-square (8) = 4.92, *p* = 0.766, area under the receiver operating characteristic (ROC) curve = 71.1 (95% CI = 58.4–83.8).

**Table 8:** Patients’ clinicopathological characteristics based on the IGF-1 status (*n* = 75).

Clinicopathological Characteristics	IGF-1 Expression	
	Negative (*n* = 8)	Positive (*n* = 67)
**Histological Grade**		
Grade 1	0 (0.0%)	10 (14.9%)
Grade 2	2 (25.0%)	37 (55.2%)
Grade 3	6 (75.0%)	20 (29.9%)
**Tumor Size**		
<5 cm	3 (37.5%)	27 (40.3%)
>5 cm	5 (62.5%)	38 (56.7%)
Unknown	0 (0.0)	2 (3.0)
**Lymph Node**		
Negative	2 (25.0)	15 (22.4)
Positive	6 (75.0)	49 (73.1)
Unknown	0 (0.0)	3 (4.5)
**ER Status**		
Positive	2 (25.0%)	40 (59.7%)
Negative	6 (75.0%)	27 (40.3%)
**PR Status**		
Positive	1 (12.5%)	35 (52.2%)
Negative	7 (87.5%)	32 (47.8%)
**HER2 Status**		
Negative (0, 1+, 2+)	7 (87.5%)	46 (68.7%)
Positive (3+)	1 (12.5%)	21 (31.3%)
**Molecular Subtype**		
Non-Triple–negative	3 (37.5%)	52 (77.6%)
Triple-negative	5 (62.5%)	15 (22.4%)

**Table 9:** Multiple logistic regression analysis of the clinicopathological predictors of IGF-1 expression (*n* = 75).

Clinicopathological Predictors	Adjusted OR (95% CI)	*p*-Value
**Histological Grade**		
Grade 1	-	
Grade 2	10.92 (1.39, 255.51)	0.052
Grade 3	1	
**Tumor Size**		
<5 cm	1	
>5 cm	2.6 (0.25, 33.21)	0.420
**Lymph Node**		
Negative	1	
Positive	0.46 (0.02, 5.7)	0.573
**ER Status**		
Positive	1	
Negative	0.77 (0.01, 44.59)	0.895
**PR Status**		
Positive	1	
Negative	0.07 (0, 2.13)	0.129
**HER2 Status**		
Negative (0, 1+, 2+)	1	
Positive (3+)	13.78 (0.41, 1626.39)	0.209
**Molecular Subtype**		
Non-Triple-Negative	1	
Triple-Negative	3.46 (0.05, 294.25)	0.561

Model fitness for multiple logistic regression analysis: Hosmer–Lemeshow chi-square (8) = 4.13, *p* = 0.845, area under the receiver operating characteristic (ROC) curve = 85.2 (95% CI = 75.1–95.2).

## Data Availability

All data present within the main article.
